# Socioeconomic inequalities and health behaviours in depression: a picture of mental health in Portugal

**DOI:** 10.1093/eurpub/ckag087

**Published:** 2026-07-03

**Authors:** Maria R Paiva Pessoa, Joana Alves, Cristiana Duarte, Carolina Reis, Amélia Nunes, João Pereira

**Affiliations:** NOVA National School of Public Health, Public Health Research Centre, Comprehensive Health Research Center, CHRC, REAL, CCAL, NOVA University Lisbon, Lisbon, Portugal; NOVA National School of Public Health, Public Health Research Centre, Comprehensive Health Research Center, CHRC, REAL, CCAL, NOVA University Lisbon, Lisbon, Portugal; NOVA National School of Public Health, NOVA University Lisbon, Lisbon, Portugal; NOVA National School of Public Health, NOVA University Lisbon, Lisbon, Portugal; NOVA National School of Public Health, NOVA University Lisbon, Lisbon, Portugal; University of Beira Interior, Covilhã, Portugal; NOVA National School of Public Health, Public Health Research Centre, Comprehensive Health Research Center, CHRC, REAL, CCAL, NOVA University Lisbon, Lisbon, Portugal

## Abstract

Depressive disorders represent the second leading cause of disability globally, and Portugal reports the second highest prevalence in Europe. Nevertheless, the role of socioeconomic factors, behavioural determinants, and potential differences in treatment remain underexplored. This study assessed socioeconomic inequalities in depression and inequity in mental healthcare utilization among Portuguese adults aged 25–65 years, and evaluated whether health behaviours mediate the socioeconomic status (SES) and depression association. We used microdata from the 2019 Portuguese National Health Interview Survey. Depression was measured through self-report and PHQ-8 (≥10, moderate and moderately-severe; ≥20, severe). Concentration curves and indices, standardized by sex and age, assessed SES-related inequality in depression; horizontal inequity in mental healthcare utilization was estimated by adjusting for morbidity. Logistic regression models estimated the SES-depression association, and mediation by health behaviours (smoking, alcohol, sedentary lifestyle, diet, BMI) was evaluated using attenuation analysis. Overall, 13.0% reported depression in the previous year and 6.8% met PHQ-8 criteria. Both were disproportionately concentrated among lower-income groups, with the strongest inequality observed for severe depression. Horizontal inequity was also observed: specialist consultations were disproportionately used by higher-income groups when adjusting for self-reported depression, whereas medication was more concentrated among lower-income individuals meeting PHQ-8 criteria. Sedentarism, BMI, and alcohol drinking partially mediated SES-depression association, reducing effect estimates by up to 23.9%. Marked socioeconomic inequalities exist in depression and mental healthcare utilization in Portugal. Strengthening equitable access to evidence-based mental healthcare and addressing upstream behavioural and socioeconomic determinants are critical to reducing the national mental health burden.

## Introduction

Depressive disorders are characterized by long periods of low mood, loss of pleasure, or lack of interest in activities. These conditions arise from a complex interplay of social, psychological, environmental, and biological factors [1]. The latest *Global Burden of Disease* studies estimate depressive disorders as the second leading cause of years of life with disability (YLDs), having reached 56.3 million YLDs in 2021 [2].

Crucially, individuals from a lower socioeconomic status (SES) are disproportionally affected by depression [3, 4]. Financial strain, material deprivation, and lower education levels increase the risk of depression and are associated with reduced access to treatment, particularly mental health consultations [5, 6]. A review reported that 10 of 11 studies identified a statistically significant association between the prevalence of common mental disorders and markers of lower SES [7]. Furthermore, lower SES is associated with worse prognoses and poorer treatment outcomes [8]. Across the European Union, low-income households are five times more likely to report unmet medical care needs than high-income households, suggesting observed unmet care for depression is more concentrated among the poorest [9].

Beyond socioeconomic disparities in prevalence and treatment, lifestyle factors may further contribute to a heavier mental health burden. Unhealthy behaviours, such as poor diet, sedentary lifestyle, smoking, and alcohol consumption, have been shown to be associated with, and potentially contribute to, the development of mental health issues [10–13]. Conversely, adoption of healthy behaviours can promote overall well-being and enhance treatment outcomes, especially when combined with conventional medical interventions [10, 14]. Many of these unhealthy behaviours are more common among lower SES groups, exacerbating health inequalities and contributing to disparities in mortality [15].

The public health challenge of poor mental health is particularly pressing in Portugal. In 2016, 9.3% of individuals registered in primary healthcare services (within the public National Health Service) were diagnosed with depression, more than double the current WHO global estimates (see Supplementary References). Estimates of depression prevalence in 27 European countries place Portugal as the second highest in Europe, surpassed only by Iceland [16]. In 2017, only 18.8% of individuals diagnosed with major depressive disorder received minimally adequate treatment. The comparable average figure for high-income countries is 22.4% [17].

Despite these concerns, the interplay between SES, depression and health behaviours in the Portuguese population has yet to be adequately explored. In this study, using the latest National Health Interview Survey data (2019), we aimed to examine first, socioeconomic inequalities in depression, using self-reported measures of prevalence and the Patient Health Questionnaire (PHQ-8), second, inequity in mental health treatment, including unmet care need, and third, the role of health behaviours in the relationship between SES and depression.

## Methods

### Data and study design

Cross-sectional microdata was obtained from the 2019 Portuguese National Interview Survey [part of the European Health Interview Survey (EHIS)], a representative, probabilistic, stratified and multistage sample of non-institutionalized residents aged 15 or older (*N* = 14 617). This is a population-based survey carried out approximately every 5 years, with the 2019 wave being the most recently available. Data were collected through computer-assisted personal interviewing (CAPI) and computer-assisted web interviewing (CAWI) [18, 19]. We focused our analysis on working-age groups (25–64 years) to exclude potential effects of comorbidities on depression, which are highly correlated with advanced age, and to avoid selective mortality bias [20]. Data were excluded where missing or no response was observed. Sample size for this study was 7901 individuals. This study used anonymized microdata, made available by Statistics Portugal (INE); ethical approval was not required.

### Variables

Age was used in the analyses grouped in 5-year intervals, from 25 to 64 years old. Household disposable income per adult equivalent, using the OECD modified scale, was used as the main SES variable [21]. Data were collected using income intervals and made available to researchers, grouped as approximate income quintiles.

We used two different methods of assessing depression in our sample. Firstly, we used a self-reported measure, based on respondents replying positively to the question ‘Suffers from or has suffered from depression’ (previous 12 months). Secondly, we used the Patient Health Questionnaire-8 (PHQ-8), a widely used and validated tool for depressive disorder, referring to the previous 2-week period (scores 0–3: not at all, several days, more than half the days, nearly every day), yielding a total score from 0 to 24. We classified participants according to those scoring 10 points or higher [classified as having moderate to moderately-severe depressive symptoms (clinically depression)] and 20 points or higher (severe symptoms) [22]. In addition, we examined inequality in the eight specific items of the PHQ-8, where a positive response (i.e. experienced the situation for several days, more than half the days or nearly every day) was the cut-off considered for the inequality analyses.

To measure access to mental health treatment, three binary variables were included: ‘Consultation with a psychologist, psychotherapist or psychiatrist’ (hereafter referred to as Mental health specialist consultation), ‘Regular use of medication for depression, prescribed by a physician’ (Prescribed medication for depression), and ‘Unmet need for a mental health consultation or treatment due to financial difficulties’ (Unmet mental health care need), all reported to the previous 12 months.

A comprehensive description of how health-related behaviour variables were operationalized and dichotomized, including sedentary lifestyle, alcohol consumption, smoking status, dietary index, and body mass index (BMI), is available in Supplementary Methods.

### Statistical analyses

Concentration curves for mental health variables were calculated and plotted against a line of equality, by calculating point estimates (linear approximation method) of the cumulative percentage of the health variable against the cumulative percentage of the sample, ranked by income quintile, from lowest to highest [23]. These were adjusted for sex and age using the direct method, by applying age- and sex-specific rates to the overall sample distribution as the standard population, thus obtaining standardized estimates. For healthcare utilization variables, we further adjusted for morbidity (self-reported and PHQ-8-scored depression), to provide concentration curves and indices of horizontal inequity [24]. Furthermore, concentration indices for these health variables were calculated, with their respective 95% confidence intervals, using income quintiles as the living standards variable. The concentration index quantifies the degree of socioeconomic inequality in health variables, calculated as twice the area between the concentration curve and the line of equality. Values closer to zero indicate more equal distribution, while negative values reflect a stronger concentration of the health/utilization variable among the poorest groups [23].

Binomial logistic regression models were used to assess the association between SES (income quintiles) on both self-reported diagnosis and PHQ-8 scored depression, adjusted for sex and age (baseline SES model). To evaluate the potential mediating role of individual health behaviours (HB) and BMI, each variable was added separately to the baseline SES model. The proportion of the SES effect on depression mediated by each HB was estimated using the formula described in Stringhini *et al.*: (β_Model SES_ − β_Model SES + HB_)/β_Model SES_ × 100, where β_Model SES_ is the coefficient for the 1st income quintile in the baseline model, using the 5th income quintile as reference, and β_Model SES + HB_ is the coefficient after adding the health behaviour [15]. To estimate the 95% confidence intervals (95% CI) for the estimated proportion of the mediating role of each health behaviour, a bias-corrected and accelerated bootstrapping method, with 3000 resamples, was used. Statistical significance was set at *P < .*05 and analyses were conducted using Stata (SE version 13.0) and R (R studio Version 2024.12.1 + 563).

## Results

We analysed data from a sample of 7901 people aged 25 to 64 years old who participated in the last National Health Interview Survey in Portugal, in 2019, corresponding to 54.1% of the total survey sample. Some analyses required a smaller sub-sample due to missing data; a detailed characterization of the study samples is presented in Table 1 and Supplementary Fig. S1.

**Table 1.** Sample characteristics: 25- to 64-year-old individuals who participated in the 2019 Portuguese National Health Interview Survey

	* n*	%	95% CI
Sex			
Men	3519	44.5	43.4–45.6
Women	4382	55.5	54.4–56.6
Age groups			
25–29	483	6.1	5.6–6.7
30–34	582	7.4	6.8–8.0
35–39	830	10.5	9.8–11.2
40–44	1082	13.7	12.9–14.5
45–49	1094	13.8	13.1–14.6
50–54	1154	14.6	13.8–15.4
55–59	1309	16.6	15.8–17.4
60–64	1367	17.3	16.5–18.1
Income quintiles			
1st quintile	1700	21.5	20.6–22.4
2nd quintile	1192	15.1	14.3–15.9
3rd quintile	1554	19.7	18.8–20.6
4th quintile	1781	22.5	21.6–23.5
5th quintile	1674	21.2	20.3–22.1
Education levels (ISCED 2011)			
Low (0–2)	4362	55.2	54.1–56.3
Middle (3–4)	1795	22.7	21.8–23.7
High (5–8)	1744	22.1	21.2–23.0
Depression			
Self-reported—12-month rp	1030	13.0	12.3–13.8
Depression (PHQ-8 ≥ 10)—2-week rp	536	6.8	6.2–7.4
Severe depression (PHQ-8 ≥ 20)—2-week rp	78	1.0	0.8–1.2
Mental healthcare utilization^a^			
Prescribed medication for depression	927	11.8	11.1–12.5
Mental health specialist consultation	623	7.9	7.3–8.5
Unmet mental health care need	246	3.1	2.8–3.5
Health behaviours^b^			
Sedentary lifestyle	5531	72.7	71.7–73.7
Unhealthy diet	1320	17.4	16.5–18.2
Drinks alcohol three to four times per week or more often	3149	41.4	40.3–42.5
Smokes daily	1580	20.80	19.90–21.70
Obesity (BMI ≥30 kg/m^2^)	1452	18.8	17.9–19.6

rp, reference period.

a Sub-samples of the population *n* = 7858—please see Supplementary Fig. S2.

b Sub-samples of the population *n* = 7602—please see Supplementary Fig. S2.

Over half of participants were women (55.5%) and belonged to older age groups (62.3% >45 years). Educational attainment was predominantly low, with 55.2% of respondents having completed only lower levels of education (from no formal education up to lower secondary school), as classified by International Standard Classification of Education (ISCED) 2011, widely used in the EU. Overall, 13.0% of individuals self-reported depression over the previous 12 months, while 6.8% met the criteria for depression based on the PHQ-8 scale (score ≥10), and 1.0% met the threshold for severe depression (score ≥20), in a 2-week reference period. This is consistent with OECD 2019 data, which indicate that, on average, 4.9% of Portuguese men and 9.8% of Portuguese women aged ≥18 years reported moderate-to-severe depressive symptoms [25]. Use of mental health services was reported by 11.8% for medication for depression and 7.9% for specialist consultations, while 3.1% reported an unmet clinical need for mental healthcare due to financial barriers. Health behaviours revealed a high prevalence of sedentary lifestyle (72.7%), while 17.4% reported having an unhealthy diet. One-fifth of participants smoked daily (20.8%), and 41.4% reported consuming alcohol at least three to four times per week. Obesity affected nearly one in five participants (18.8%).

### Depression inequality in Portugal

**Figure 1.**
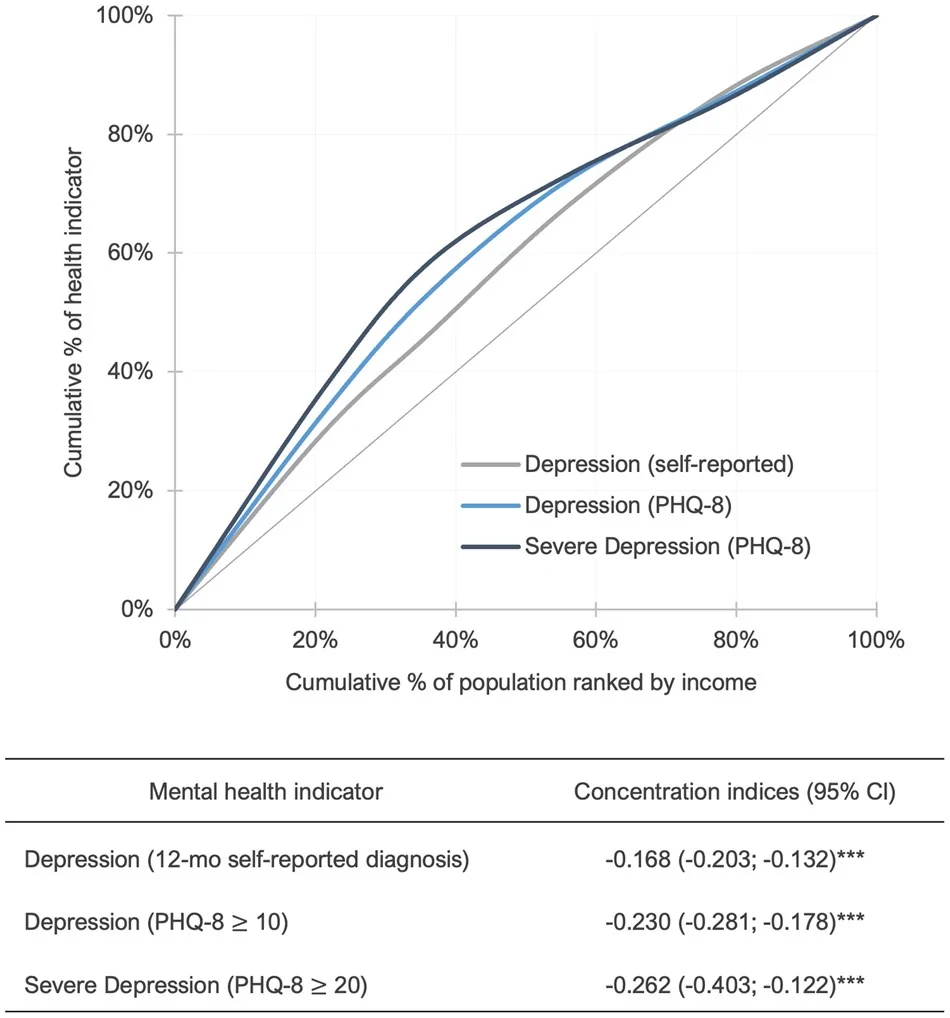
Concentration curves and indices for depression in Portugal, measured by self-reported (previous 12 months) and the PHQ-8 responses, above 10 points and 20 points, referring to the previous 2 weeks. 95% CI, 95% confidence intervals. *** *P* < .001.

To assess the level of inequality in depression in the Portuguese working-age population, both 12-month prevalence and 2-week PHQ-8 scored concentration curves were plotted, adjusted for sex and age, and ranked by income quintile (Fig. 1).

Concentration curves and indices for depression revealed marked inequality of this illness among the Portuguese working-age population, disproportionally affecting the lowest income quintiles. Severe depression showed the largest deviation from equality, with a concentration index of −0.262 (95% CI: −0.403 to −0.122), while self-reported depression over the last 12 months appeared closer to equality (95% CI: −0.203 to −0.132).

Next, we sought to assess inequality in each item of the PHQ-8, to help elucidate how specific symptoms associated with depressive disorder are distributed per income quintile. All eight items included in the PHQ-8 were significantly more concentrated in the most disadvantaged groups, ranging from −0.209 (95% CI: −0.242 to −0.176—‘Feeling bad about yourself (…)’) to −0.052 (95% CI: −0.070 to −0.039—‘Feeling tired or having little energy’), providing evidence that all items in this questionnaire disproportionally affect the poorest (Supplementary Table S1).

### Horizontal inequity in mental healthcare access

**Figure 2.**
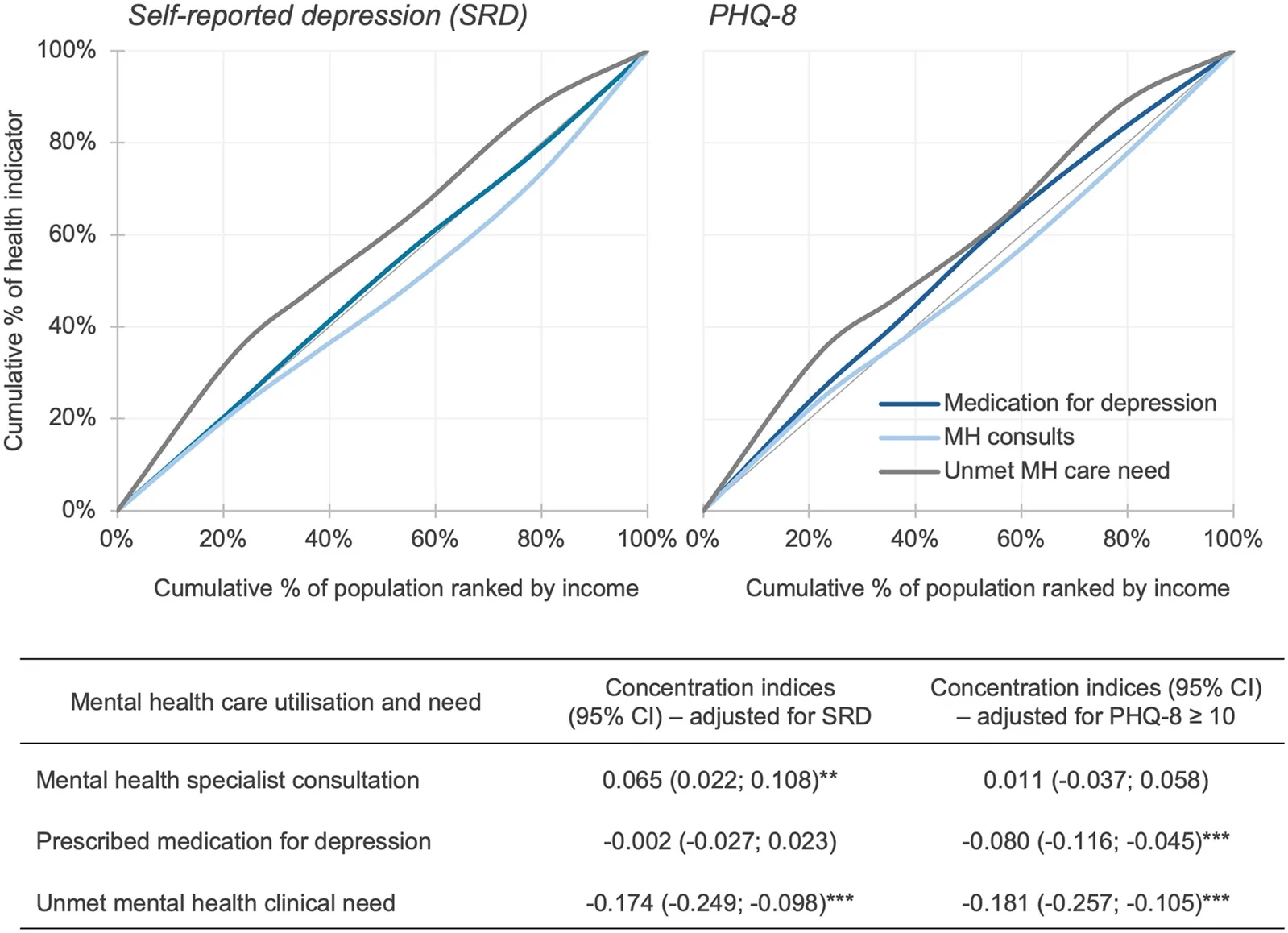
Concentration curves and indices, adjusted for sex, age, and morbidity, for mental health treatments and unmet mental health clinical need in Portugal, referring to the previous 12 months. **P < .01 ***P < .001. 95% CI, 95% confidence intervals.

Concentration indices for mental health specialist consultations revealed pro-rich horizontal inequity for self-reported depression (0.065, *P < .*01), but no horizontal inequity was observed for PHQ-8-scored depression (0.011, ns) Conversely, we observed horizontal inequity in medication for depression favouring the poorest in PHQ-8 scored depression (−0.080, *P < .*001), while no inequity was observed in the self-reported adjusted curve and index (−0.002, ns). Unsurprisingly, unmet mental health clinical need revealed marked horizontal inequity favouring the rich when adjusted for both health outcomes (Fig. 2).

### Health behaviours in the association between SES and depression

Next, we examined the distribution of health-related behaviours across income quintiles (Supplementary Table S2). Obesity was significantly more prevalent among those in the lower-income quintiles, with nearly one in four individuals classified as obese, compared to 13.6% in the highest quintile. Similar trends were seen in dietary patterns. Sedentary lifestyle followed the most striking gradient: over 80% of individuals in the lowest quintiles reported insufficient activity, compared to just 55.6% in the highest. Conversely, alcohol consumption patterns deviated from this overall trend: higher frequency in alcohol consumption was more prevalent among higher-income groups, indicating a reverse social gradient. Notably, a greater proportion of individuals in lower-income groups identified as former drinkers. Smoking patterns revealed interesting disparities; while lowest income groups had a higher proportion of non-smokers, they also exhibited higher daily smoking rates compared to the richest groups. This difference was primarily explained by a larger proportion of former smokers in the top-income quintiles, in contrast to what we observed with alcohol.

We then evaluated the association between depression and income quintiles, while estimating the mediating effect of health behaviours. Table 2 presents the associations between the lowest household income quintile and depression, identified through 12-month self-reported and 2-week PHQ-8 ≥10, using binomial logistic regression models adjusted for sex and age. In the baseline model (SES), individuals in the lowest income quintile had significantly higher odds of depression compared to those in the highest quintile, with adjusted odds ratios (AOR) of 2.68 (95% CI: 2.14–3.37, *P < .*001) for self-reported depression, and 2.70 (95% CI: 2.01–3.65, *P < .*001) for PHQ-8 ≥10 points.

**Table 2.** Association between socioeconomic status (income quintiles) and depression, both self-reported and PHQ-8-scored (≥10) and estimated mediating effect of each individual health behaviour (HB), as well as the fully adjusted model, with all health behaviours, and BMI, included

Self-reported depression (12 months)	β_SES_	AOR_SES_ (95% CI)	Sig.	
1st income quintile (SES baseline)	0.985	2.68 (2.14 to 3.37)	***	–
	β_SES + HB_	AOR_SES + HB_ (95% CI)		Attenuation (95% CI)
+ Drinking alcohol	0.915	2.50 (1.99 to 3.15)	***	7.12% (4.37 to 10.70)
+ Unhealthy diet	0.983	2.67 (2.14 to 3.36)	***	0.16% (−0.82 to 1.13)
+ Unhealthy diet and BMI	0.911	2.49 (1.98 to 3.13)	***	7.54% (4.47 to 11.43)
+ Sedentary lifestyle	0.870	2.39 (1.90 to 3.01)	***	11.73% (7.15 to 17.56)
+ Sedentary lifestyle and BMI	0.812	2.25 (1.79 to 2.85)	***	17.89% (12.00 to 25.14)
+ BMI	0.912	2.49 (1.99 to 3.14)	***	7.38% (4.44 to 11.24)
+ Smoking	0.988	2.69 (2.15 to 3.38)	***	−0.32% (−1.94 to 1.04)
Fully adjusted	0.752	2.12 (1.68 to 2.69)	***	23.92% (16.70 to 33.05)

PHQ-8 ≥ 10 (2 weeks)	β_SES_	AOR_SES_ (95% CI)	Sig.	
1st income quintile (SES baseline)	0.992	2.70 (2.01 to 3.65)	***	–
	β_SES + HB_	AOR_SES + HB_ (95% CI)		Attenuation (95% CI)
+ Drinking alcohol	0.923	2.52 (1.88 to 3.41)	***	6.97% (3.58 to 11.24)
+ Unhealthy diet	0.998	2.71 (2.03 to 3.67)	***	−0.62 % (−2.13 to 0.53)
+ Unhealthy diet and BMI	0.938	2.56 (1.90 to 3.47)	***	5.48 % (1.45 to 10.66)
+ Sedentary lifestyle	0.915	2.50 (1.86 to 3.40)	***	7.80% (2.09 to 14.79)
+ Sedentary lifestyle and BMI	0.867	2.38 (1.77 to 3.24)	***	12.84% (6.12 to 22.15)
+ BMI	0.933	2.54 (1.89 to 3.45)	***	6.11% (2.67 to 11.13)
+ Smoking	0.991	2.69 (2.01 to 3.65)	***	−0.01% (−1.67 to 1.52)
Fully adjusted	0.807	2.24 (1.66 to 3.06)	***	18.95% (10.52 to 29.75)

Coefficients (β) correspond to 1st income quintile (SES), using the 5th income quintile as reference.

95% CI, confidence interval; AOR, adjusted odds ratio; SES, socioeconomic status; Sig., level of statistical significance.

*** *P* < .001.

To explore potential mediation of health behaviours, each was added individually to the baseline model, and then combined into the fully adjusted model for each outcome. The mediating effect was calculated as the percentage reduction in the association between income-depression (attenuation), using the regression’ coefficients. Sedentary lifestyle and BMI were associated with the largest individual mediating effects, while having an unhealthy diet and smoking showed no significant mediating impact, both in self-reported and PHQ-8 scored depression. In the fully adjusted models, including all behaviours and BMI, the income-depression association was attenuated by 23.92% (95% CI: 16.70–33.05) for self-reported depression and 18.95% (95% CI: 10.52–29.75) for PHQ-8 scored depression, suggesting these factors may play a partial mediating role in socioeconomic disparities in this disease (Table 2).

## Discussion

This study identified socioeconomic inequalities in depression and horizontal inequity in prescribed medication, mental health specialist consultations, and unmet mental health needs due to financial barriers in a representative sample of Portuguese adults aged 25–64 in 2019. Furthermore, we estimated health behaviours, such as alcohol, smoking, unhealthy diet, and sedentary lifestyle, and BMI, may partly mediate the association between depression and low income. To our knowledge, this is the first study to show this association and inequality in depression and horizontal inequity in mental healthcare in Portugal.

Overall, 13.0% of participants reported current or past depression in the previous 12-months, while only 6.8% met the 2-week PHQ-8 criteria. The difference between the two rates reflects what each measures; self-reported depression may include people who have been diagnosed and have recovered, or are undergoing treatment (successful or not), whereas PHQ-8 scored depression provides a short-term frequency of depressive disorder symptoms, which may not have been diagnosed, nor treated, or that result from ineffective treatment [22]. Subclinical depression and life events that lead to a temporary depressed mood (grief, medical condition/injury, unemployment, among others) could be captured differently by the various instruments to measure depressive disorder and explain variation of estimates in the literature [26]. Nevertheless, depression remains a substantial and growing burden in Portugal, with NHS primary care clinical diagnosis nearly doubling from 2011 to 2016 (5.3% to 9.3%) (see Supplementary References).

Depression was concentrated among lower-income groups for both self-reported and PHQ-8 measures, consistent with international evidence [4–6]. PHQ-8 scored depression revealed marked inequality regarding overall prevalence and active symptoms.

Medication use for depression was reported by 11.8%, and 7.9% had consulted a mental health specialist, although consultation frequency was not captured. Antidepressant consumption has risen sharply across Europe, with Portugal currently at 154 defined daily doses per 1000 inhabitants per day, *albeit* not directly comparable with our data [27]. This rise may reflect prolonged use of medications and new therapeutic indications, but also growing demand amid limited specialist capacity for non-pharmacological interventions [28]. In 2020, Portugal had 20.96 mental health professionals per 100 000 people, including private providers, well below the European average of 44.8, while the estimated number of psychologists working within the Portuguese NHS was 8.5 and 7.8 psychiatrists per 100 000 people [29, 30]. Limited public-sector supply is particularly relevant to our findings; specialist consultations favoured high-income groups when adjusting for self-reported depression, whereas no inequity was observed for PHQ-8 depression. This pattern suggests people with active symptoms, regardless of a formal diagnosis and SES, may have comparable access to a specialist; however, since frequency and context of consultations is lacking, it is possible a significant proportion of these could be acute episodes (e.g. emergency episodes), rather than continued therapeutic treatment, which is mostly available through private providers, requiring out-of-pocket payment, and not often covered by insurance. Conversely, prescribed medication showed no inequity among those with a self-reported diagnosis but favoured lower-income individuals with active symptoms. This suggests pharmacological treatment prescribed in primary care is likely the main avenue for managing depression in lower-SES groups, whereas high-income groups may favour specialist consultations, mostly through private providers, without the existing gate-keeping of the NHS [28].

Despite rising depression rates and the need to improve quality of treatment being recognized in the latest Portuguese Mental Health Programme (2017), current national guidelines (2012) focus solely on severe cases and its pharmacological treatment (Supplementary References). In contrast, the UK’s National Institute for Health and Care Excellence (NICE) recommends antidepressants should not be prescribed as first-line treatment in moderate disease (unless by patient preference) but rather offer several options of evidence-based therapy interventions [31].

Our findings on income distribution of mental health-associated behaviours align with previous reports; socioeconomically disadvantaged groups are more likely to engage in unhealthier behaviours [32, 33]. However, more frequent alcohol consumption was more prevalent among higher-income respondents, consistent with findings from other countries. Because the available data do not distinguish between social ‘moderate’ drinking and alcohol dependency/harmful binge drinking, which are associated with poorer mental health, the interpretation of this reverse social gradient requires caution [34]. Our results show partial mediation of physical activity (and BMI), but not smoking or unhealthy diet, in the relationship between low SES-depression, consistent with other longitudinal studies focused on physical health and mortality [15, 35]. Here, we did not aim to provide a causal explanation for this mediation; rather, we aimed to provoke hypotheses of plausible mechanisms, requiring further investigation, on behavioural determinants of health. Some pathways can be considered when interpreting these results; positive effects of physical activity on mental health have been extensively studied, and binge-drinking (rather than frequency) is known to increase the risk of mental ill-health [36, 37]. Interestingly, a causal link between lifetime smoking and depression has been proposed, through an inflammatory marker, although we did not find a mediation role in our analyses [13]. The plausible effect of an unhealthy diet is much less direct, and we did not find a significant mediation role; it would most likely be associated with depression through physical ill-health, although there is some evidence of association between ultra-processed foods and mental health disorders [38]. Taken together, evidence suggests that low SES individuals, who are more vulnerable to unhealthy behaviours, also become more at-risk of depressive disorders partly due to these behaviours, particularly sedentarism.

### Limitations

This study has several limitations. Its cross-sectional design prevents causal inference between SES-depression and behaviour, as well as trends on inequities here identified. Information on mental healthcare use was also restricted, particularly regarding the type and frequency of specialist consultations, constraining the interpretation of treatment patterns underlying the observed inequities. Notably, we used two different measures of depression in this study, which are not intended to replace diagnosis but to indicate potential depressive symptomatology. As noted, these capture distinct aspects of depression, and even though PHQ-8 is an imperfect tool, potentially overestimating true population prevalence, these findings are consistent with the latest clinical diagnoses estimates, supporting the validity of the results [22]. Additionally, we were not able to distinguish between forms of alcohol consumption (i.e. social vs. harmful drinking), which limited mediation estimates. We were also unable to explore regional differences in inequalities, an important dimension given Portugal’s known geographic variation in healthcare provision. Lastly, available data are from 2019, and patterns of depression and mental healthcare utilization may have changed since then, particularly as the COVID-19 pandemic posed a major challenge to mental health [9]; replicating these analyses with data from subsequent survey waves would allow assessment of post-pandemic trends. Despite these limitations, the study provides robust evidence on socioeconomic disparities in depression and mental healthcare utilization in Portugal, using validated measures and established inequality metrics. It also highlights meaningful behavioural pathways that can inform preventive strategies.

### Policy implications

Our findings highlight the pressing need for targeted public policies aimed at reducing socioeconomic inequalities in depression and enhancing equity in mental healthcare provision in Portugal. The persistent shortage of mental health professionals, combined with the clinical complexity of depression management, pose significant system-level challenges which are difficult to overcome [39]. While evidence underscores the pivotal role of primary healthcare (PHC) in prevention and treatment of mild mental illness, the existing PHC pay-for-performance incentives in Portugal continue to exclude mental health indicators, limiting their capacity to address the burden with the served population. A comprehensive model of mixed payment schemes to be implemented in PHC has already been proposed and validated by a panel of experts, which could serve as a stepping stone [40].

Consequently, public policies addressing upstream social determinants of health, such as disproportionate exposure of vulnerable groups to unhealthy behaviours, may help prevent additional disease burden and mitigate observed inequalities. In parallel, the revision of national clinical guidelines for depression, informed by further analyses of SES inequalities and inequities in treatment, as well as potential mediating mechanisms, represent an urgent policy priority towards achieving equitable and effective care. Our findings lay important groundwork for future longitudinal research, offering a valuable contribution to national mental health policy efforts.

## Conclusion

In summary, this study provides robust evidence of marked socioeconomic inequalities in both depression and mental healthcare utilization in Portugal. By highlighting behavioural mediators and treatment inequities, it identifies clear opportunities for policy action aimed at improving mental health outcomes. Future research should examine longitudinal trends to better understand the drivers of the observed inequities, particularly in the post-pandemic context, as well as regional disparities in disease burden and access to mental healthcare.

## Supplementary Material

ckag087_Supplementary_Data

## Data Availability

Data from the Portuguese National Health Interview Survey are owned by Statistics Portugal (INE) and are available upon formal request through the INE microdata access procedure at www.ine.pt. The authors will make the analysis code available upon reasonable request. Key points Depression in Portugal is disproportionately concentrated among lower-income adults. Socioeconomic inequities were also evident in mental healthcare use, with higher-income groups more likely to access specialist care and lower-income groups relying more on pharmacological treatments. Health behaviours, particularly sedentary lifestyle, BMI and alcohol consumption, partially mediated the SES-depression association, suggesting modifiable pathways for prevention and burden of disease mitigation. Findings highlight the need to strengthen equitable access to evidence-based mental healthcare. Public health policies addressing behavioural and socioeconomic determinants of mental health are essential for narrowing mental health inequalities and contributing to reduce disease burden in Portugal.
