# Supplementary material for: Socioeconomic inequalities and health behaviours in depression: a picture of mental health in Portugal
Source: Eur J Public Health. 2026 Jul 3;36(4):ckag087. doi: 10.1093/eurpub/ckag087 (PMC13330925; doi:10.1093/eurpub/ckag087)
Supplement: ckag087_Supplementary_Data [file ckag087_supplementary_data.zip › ejph-2026-01-om-0069-File007.docx]

**Table S1.** Concentration indices of each of the PHQ-8 items, answering “Several days” or more often to the question “Over the last two weeks, how often have you been bothered by any of the following problems?”. Standardised for sex and age. *** p<0.001. 95% CI – confidence interval.

| PHQ-8 items – “Several days” or more often | | Concentration indices  (95% CI) |
| --- | --- | --- |
| 1 | little interest or pleasure in doing things | -0.107 (-0.133; -0.081)*** |
| 2 | feeling down, depressed, or hopeless | -0.155 (-0.133; -0.081)*** |
| 3 | trouble falling or staying asleep, or sleeping too much | -0.059 (-0.181; -0.130)*** |
| 4 | feeling tired or having little energy | -0.052 (-0.070; -0.039)*** |
| 5 | poor appetite or overeating | -0.124 (-0.160; -0.089)*** |
| 6 | feeling bad about yourself, or that you are a failure, or have yourself or your family down | -0.209 (-0.242; -0.176)*** |
| 7 | trouble concentrating on things, such as reading the newspaper or watching television | -0.181 (-0.218; -0.143)*** |
| 8 | moving or speaking so slowly that other people could have noticed (or the opposite) | -0.196 (-0.244; -0.147)*** |
