# Supplementary material for: Socioeconomic inequalities and health behaviours in depression: a picture of mental health in Portugal
Source: Eur J Public Health. 2026 Jul 3;36(4):ckag087. doi: 10.1093/eurpub/ckag087 (PMC13330925; doi:10.1093/eurpub/ckag087)
Supplement: ckag087_Supplementary_Data [file ckag087_supplementary_data.zip › ejph-2026-01-om-0069-File008.docx]

**Table S2.** Distribution of sample’s health behaviour characteristics per income quintile (n = 7,602).

|  | 1st quintile | | | | 2nd quintile | | | | 3rd quintile | | | 4th quintile | | | | | 5th quintile | | | | |  |
| --- | --- | --- | --- | --- | --- | --- | --- | --- | --- | --- | --- | --- | --- | --- | --- | --- | --- | --- | --- | --- | --- | --- |
|  | n | % (95% CI) | | | n | % (95% CI) | | | n | % (95% CI) | | | | n | % (95% CI) | | | | n | % (95% CI) | |  |
| Normal or underweight | 650 | | 40.1% (37.8%; 42.5%) | 478 | | | 41.9% (39.1%; 44.8%) | 590 | | | 39.1% (36.8%; 41.7%) | | 754 | | | 43.9% (41.6%; 46.3%) | | 803 | | | 49.6% (47.1%; 52.0%) | |
| Overweight | 598 | | 36.9% (34.5%; 39.2%) | 444 | | | 38.9% (36.1%; 41.7%) | 600 | | | 39.9% (37.5%; 42.5%) | | 654 | | | 38.1% (35.8%; 40.4%) | | 597 | | | 36.9% (34.5%; 39.2%) | |
| Obese (BMI ≥ 30 kg/m^2^) | 373 | | 23.0% (21.0%; 25.1%) | 219 | | | 19.2% (17.0%; 21.5%) | 313 | | | 20.8% (18.8%; 22.9%) | | 309 | | | 18.0% (16.2%; 19.8%) | | 220 | | | 13.6% (12.0%; 15.3%) | |
| Healthy diet | 1310 | | 80.8% (78.9%; 82.7%) | 911 | | | 79.9% (77.5%; 82.1%) | 1252 | | | 83.3% (81.4%; 85.1%) | | 1415 | | | 82.4% (80.6%; 84.2%) | | 1394 | | | 86.0% (84.3%; 87.7%) | |
| Unhealthy diet | 311 | | 19.2% (17.3%; 21.1%) | 230 | | | 20.1% (17.9%; 22.5%) | 251 | | | 16.7% (14.9%; 18.6%) | | 302 | | | 17.6% (15.8%; 19.4%) | | 226 | | | 14.0% (12.3%; 15.7%) | |
| Physically active | 296 | | 18.2% (16.4%; 20.2%) | 217 | | | 19.0% (16.9%; 21.4%) | 320 | | | 21.3% (19.3%; 23.4%) | | 521 | | | 30.3% (28.2%; 32.5%) | | 720 | | | 44.4% (42.0%; 46.9%) | |
| Not physically active | 1325 | | 81.7% (79.8%; 83.6%) | 924 | | | 81.0% (78.6%; 83.1%) | 1183 | | | 78.7% (76.6%; 80.7%) | | 1196 | | | 69.7% (67.5%; 71.8%) | | 900 | | | 55.6% (53.1%; 58.0%) | |
| Regular alcohol drinker | 335 | | 20.7% (18.7%. 22.7%) | 276 | | | 24.2% (21.8%; 26.7%) | 441 | | | 29.3% (27.0%; 31.6%) | | 519 | | | 30.2% (28.1%; 32.5%) | | 449 | | | 27.7% (25.6%; 29.9%) | |
| Moderate alcohol drinker | 388 | | 23.9% (21.9%; 26.1%) | 331 | | | 29.0% (26.4%; 31.7%) | 427 | | | 28.4% (26.2%; 30.7%) | | 600 | | | 34.9% (32.7%; 37.2%) | | 674 | | | 41.6% (39.2%; 44.0%) | |
| Occasional alcohol drinker | 755 | | 46.6% (44.2%; 49.0%) | 455 | | | 39.9% (37.1%; 42.7%) | 534 | | | 35.5% (33.2%; 38.0%) | | 507 | | | 29.5% (27.4%; 31.7%) | | 448 | | | 27.6% (25.5%; 29.9%) | |
| Former alcohol drinker | 143 | | 8.8% (7.5%; 10.3%) | 79 | | | 6.9% (5.6%; 8.5%) | 101 | | | 6.7% (5.5%; 8.1%) | | 91 | | | 5.3% (4.4%; 6.5%) | | 49 | | | 3.1% (2.3%; 4.0%) | |
| Smokes daily | 377 | | 23.2% (21.2%; 25.3%) | 239 | | | 20.9% (18.6%; 23.4%) | 328 | | | 21.8% (19.8%; 23.9%) | | 346 | | | 20.1% (18.3%; 22.1%) | | 290 | | | 17.9% (16.1%; 19.8%) | |
| Smokes occasionally | 45 | | 2.8% (2.1%; 3.7%) | 39 | | | 3.4% (2.5%; 4.6%) | 31 | | | 2.1% (1.4%; 2.9%) | | 53 | | | 3.1% (2.3%; 4.0%) | | 61 | | | 3.8% (2.9%; 4.8%) | |
| Former smoker | 258 | | 15.9% (14.2%; 17.7%) | 222 | | | 19.4% (17.2%; 21.8%) | 333 | | | 22.1% (20.1%; 24.3%) | | 428 | | | 24.9% (22.9%; 27.0%) | | 432 | | | 26.7% (24.6%; 28.9%) | |
| Does not smoke | 941 | | 58.0% (55.6%; 60.4%) | 641 | | | 56.1% (53.2%; 59.0%) | 811 | | | 53.9% (51.4%; 56.4%) | | 890 | | | 51.8% (49.4%; 54.1%) | | 837 | | | 51.7% (49.2%; 54.1%) | |
