# Supplementary material for: Socioeconomic inequalities and health behaviours in depression: a picture of mental health in Portugal
Source: Eur J Public Health. 2026 Jul 3;36(4):ckag087. doi: 10.1093/eurpub/ckag087 (PMC13330925; doi:10.1093/eurpub/ckag087)
Supplement: ckag087_Supplementary_Data [file ckag087_supplementary_data.zip › ejph-2026-01-om-0069-File009.docx]

## Socioeconomic Inequalities and Health Behaviours in Depression:

## A Picture of Mental Health in Portugal

### Supplementary Methods

Health-related behaviour variables were dichotomised from the original variables, using a negative-threshold rationale (*i.e.* having an unhealthy diet, drinking alcohol). We included having a sedentary lifestyle in the analyses by considering individuals who reported exercising fewer than two days a week (for at least 10 minutes), below the threshold in international guidelines.[1] Regarding alcohol drinking, we considered four categories: drinking once a month to rarely or never (occasional drinking), drinking once or twice a week to once a month (moderate drinking), to reflect social drinking within the context of mental health; drinking alcohol 3-4 times a week or more (regular drinker), and the “former drinker” category was kept separate, due to possible association of prior history of addiction and related mental health issues.[2-4] To assess smoking status, a variable reporting frequency (daily, occasional or non-smoker), which also included the “former smoker” category, was used. Lastly, an unhealthy diet index (ranging from 0 to 3 points) was created, by equally weighting 3 variables: (1) eating cake, chocolates, or desserts; (2) drinking fizzy or soft drinks; and (3) eating fast food, all reported to the previous day. This index was freely based on Plichta & Jezewska-Zychowicz’s (2022) work, considering available data.[5] A 2-point threshold was defined; individuals who had a score of 2 or 3 in the newly created index were considered to have poor eating habits. Body mass index (BMI), included to adjust for potential effects of an unhealthy diet and having a sedentary lifestyle, but also assessed as an independent variable with known association with depression,[6] was calculated by dividing body weight by the square of height, and further grouped as defined by the World Obesity Federation and World Health Organisation.[7]

Supplementary References

[1] European Commission. Physical activity recommendations for adults [Internet]. Health Promotion and Disease Prevention Knowledge Gateway. Brussels: European Commission; 2021 [cited 2026 Jan]. Available from: <https://knowledge4policy.ec.europa.eu/health-promotion-knowledge-gateway/physical-activity-sedentary-behaviour-table-2b_en>

[2] Åhlin J, Hallgren M, Öjehagen A, Källmén H, Forsell Y. Adults with mild to moderate depression exhibit more alcohol related problems compared to the general adult population: a cross sectional study. BMC Public Health. 2015;15:542. doi:10.1186/s12889-015-1837-8.

[3] World Health Organization Regional Office for Europe. No level of alcohol consumption is safe for our health [news release on the Internet]. Copenhagen: WHO Europe; 2023 Jan 4 [cited 2025 Dec]. Available from: <https://www.who.int/europe/news/item/04-01-2023-no-level-of-alcohol-consumption-is-safe-for-our-health>

[4] Anderson BO, Berdzuli N, Ilbawi A, Kestel D, Kluge HP, Krech R, et al. Health and cancer risks associated with low levels of alcohol consumption. Lancet Public Health. 2023;8(1):e6–e7. doi:10.1016/S2468-2667(22)00317-6.

[5] Jezewska-Zychowicz M, Plichta M. Diet quality, dieting, attitudes and nutrition knowledge: their relationship in Polish young adults — a cross-sectional study. Int J Environ Res Public Health. 2022; 19(11):6533. doi: 10.3390/ijerph19116533.

[6] Castillo F, Francis L, Wylie-Rosett J, Isasi CR. Depressive symptoms are associated with excess weight and unhealthier lifestyle behaviors in urban adolescents. Child Obes. 2014;10(5):400–7. doi:10.1089/chi.2014.0042.

[7] World Health Organization. Obesity and overweight [fact sheet on the Internet]. Geneva: WHO; 2025 [cited 2025 Dec]. Available from: <https://www.who.int/news-room/fact-sheets/detail/obesity-and-overweight>

Additional References

Official documents from the Portuguese Directorate-General of Health (in Portuguese):

Direção-Geral da Saúde. Programa nacional para a saúde mental [National Programme for Mental Health]. Lisbon: Direção-Geral da Saúde; 2017. Available from: <https://www.dgs.pt/>

Direção-Geral da Saúde. Norma nº 034/2012 — Terapêutica farmacológica da depressão major e da sua recorrência no adulto [Guideline no. 034/2012 — Pharmacological treatment of major depression and its recurrence in adults]. Lisbon: Direção-Geral da Saúde; 2012. Available from: <https://normas.dgs.min-saude.pt/>
