# Supplementary figures and images for: Socioeconomic inequalities and health behaviours in depression: a picture of mental health in Portugal
Source: Eur J Public Health. 2026 Jul 3;36(4):ckag087. doi: 10.1093/eurpub/ckag087 (PMC13330925; doi:10.1093/eurpub/ckag087)

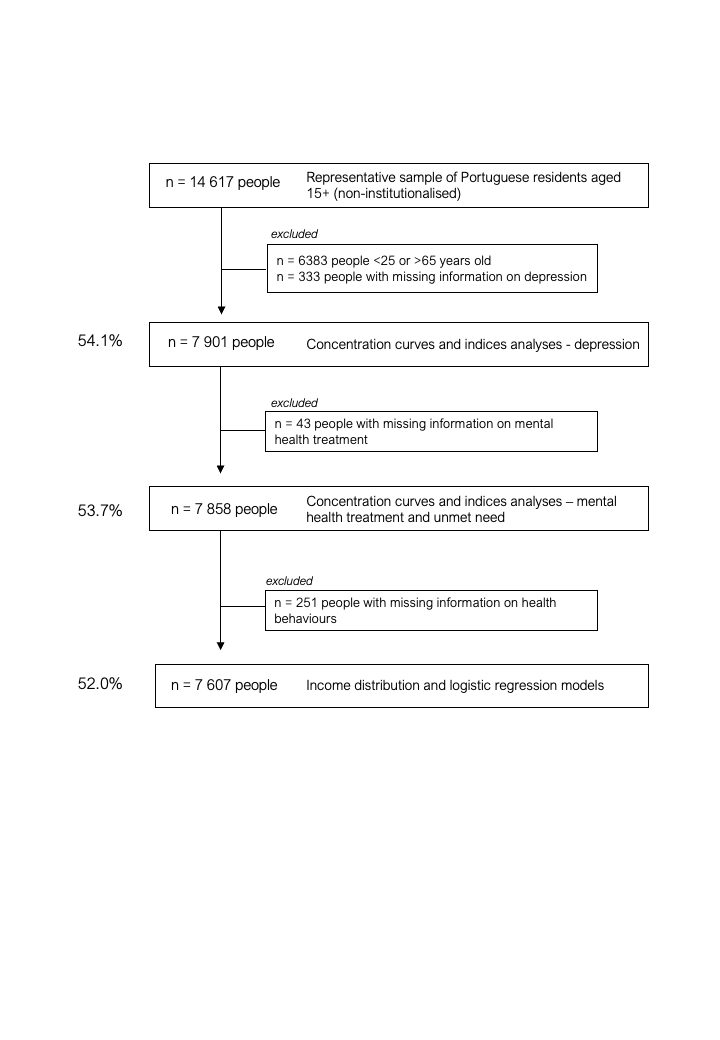

Supplement: ckag087_Supplementary_Data [file ckag087_supplementary_data.zip › ejph-2026-01-om-0069-File006.tiff]
